# Value of Left Ventricular Feature Tracking Strain Analysis for Detection of Early Cardiac Involvement in Fabry Disease (FD)

**DOI:** 10.3390/jcm10163734

**Published:** 2021-08-22

**Authors:** Fritz Christian Roller, Alexander Brose, Martin Richter, Armin Schüssler, Sebastian Harth, Christian Tanislav, Gabriele Anja Krombach

**Affiliations:** 1 Department of Diagnostic and Interventional Radiology, University Hospital Giessen, Justus-Liebig-University Giessen, Klinikstraße 33, 35392 Giessen, Germany; alexander.brose@radiol.med.uni-giessen.de (A.B.); martin.richter@radiol.med.uni-giessen.de (M.R.); armin.schuessler@radiol.med.uni-giessen.de (A.S.); sebastian.harth@radiol.med.uni-giessen.de (S.H.); gabriele.krombach@uniklinikum-giessen.de (G.A.K.); 2 Department of Neurology, University Hospital Giessen, Justus-Liebig-University Giessen, Klinikstraße 33, 35392 Giessen, Germany; christian.tanislav@diakonie-sw.de

**Keywords:** Cardiac MRI, Fabry disease, Native T1 mapping, Feature tracking strain

## Abstract

Purpose: Detection of cardiac involvement in Fabry disease (FD) is of high importance for treatment management. Native T1 mapping especially showed great potential for detection of early cardiac manifestations. Echocardiographic studies showed strain abnormalities in FD patients, but data on MRI feature tracking strain analysis (FT-SA) is limited. Therefore, the aim of our study was to evaluate the potential of FT-SA compared to native T1 and the FD specific biomarker Globotriaosylsphingosine (LysoGb3). Methods: 28 consecutive FD patients (18 female; 47.8 years ± 17.4 standard deviation (SD)) and 28 control subjects (18 female; 46.6 years ± 18.2 SD) underwent cardiac MRI at 1.5 Tesla. Global native T1 times and left ventricular FT-SA were evaluated. Results were correlated to serum Lyso-Gb3-levels. Results: Native T1 times, global longitudinal (GLS) and global radial strain (GRS) were significantly reduced in FD patients (*p* < 0.0064, *p* = 0.0009 and *p* = 0.0184, respectively). Moreover, native T1 times and GLS were significantly lower in Lyso-Gb3 positive FD patients (*p* < 0.005 and *p* = 0.03). GLS, native T1 times showed significant moderate correlations to LysoGb3 (*p* = 0.002 and *p* < 0.001). Furthermore, GLS and native T1 times reduce when LysoGb3 was elevated and increasingly with presence of left ventricular hypertrophy (LVH) or late gadolinium enhancement (LGE). Conclusions: Native T1 times and strain values differ significantly between FD patients and control subjects and showed promising correlations to the FD specific biomarker LysoGb3. We therefore conclude that strain abnormalities occur early beside native T1 reductions in cardiac FD involvement. Large scale trials are needed to verify our findings.

## 1. Introduction

Fabry disease (FD) is an X-linked disorder of lysosomal metabolism. It is characterized by accumulation of glycosphingolipids (more precisely globotriaosylceramide) in many organs (inter alia skin, myocardium and kidneys) due to a deficiency of the enzyme alpha-galactosidase [1]. Classically, male homozygotes are affected and present with burning extremity pain (acroparaesthesia) and progressive multi-organ failure in adolescence [2]. Heterozygous female carriers may present with milder disease forms compared to men [3].

Due to the storage of glycosphingolipids in cardiomyocytes, left ventricular hypertrophy (LVH) is induced, moreover, valves and vascular endothelium are also affected [4]. Later on, cardiac decompensation is triggered by myocardial fibrosis, which is usually more extensive in men than in affected women [1]. Cardiac involvement is a major factor for morbidity and mortality in FD [5].

Cardiac magnetic resonance imaging (CMRI) is well established to verify cardiac involvement. Midmyocardial inferolateral late gadolinium enhancement (LGE) without endocardial affection is a characteristic hallmark beside unspecific cardiac findings such as reduced left ventricular (LV) function and LVH [6]. In genetically confirmed FD, LGE is present in up to 50% of the patients [6]. The presence of LGE is associated with a lack in response to enzyme-replacement therapy (ERT) [7], although ERT offers good potential in reduction of LVH in patients without LGE [8] and best outcomes in early treated patients [9]. This equally underscores the diagnostic and prognostic importance of LGE in the course of FD. Conversely, LGE imaging is limited due to its dichotomous character because 15% of focal matrix expansion are required to prove LGE [10]. Hence, the benefit of LGE in FD is restricted because early cardiac involvement remains hidden and most suitable patients for ERT may not be detected by CMRI.

Native cardiac T1 mapping may solve the diagnostic dilemma. Low native T1 values are postulated to indicate accumulation of glycosphingolipids in FD and can be demonstrated in up to 59% of LVH-negative FD patients [11].

Moreover, different studies showed significant reductions of native T1 times in patients with FD [12,13,14] compared to healthy volunteers and to other cardiac diseases with LVH without any overlap [12]. A recent study showed promising correlations for native T1 times to Lyso-Gb3 [14], which is an FD specific biomarker suitable for diagnostic and monitoring [15]. In addition to native T1 times, myocardial strain analysis may serve as a further diagnostic increment because myocardial strain abnormalities in FD patients haven been shown in echocardiography studies [16,17,18,19]. However, data on CMRI-derived feature tracking strain analyses (FT-SA) in FD is limited.

FT-SA allows quantification of myocardial deformation on the basis of standardized acquired cine images without the need for additional dedicated sequences. Post myocardial infarction and in hypertrophic cardiomyopathy prediction of adverse clinical outcomes can be shown via FT-SA [20,21]. Therefore, the aim of our study is to assess the relations of FT-SA with native T1 values and the FD specific biomarker LysoGb3 in order to evaluate its diagnostic potential.

## 2. Methods

Study population: 28 consecutive and genetically confirmed enzyme replacement therapy naive FD patients (18 female) and 28 control subjects (18 female) were enrolled in this prospective cohort study from January 2014 to June 2020. 

FD diagnosis was based on a molecular genetic analysis demonstrating a heterozygous or homozygous mutation in the α-GAL-A-gene [22]. All FD patients underwent CMRI for assessment of cardiac involvement as part of their routine workup prior to therapy. To reduce magnetic field and contrast media risks for healthy volunteers, the first 28 age- and sex-matched patients, who had undergone CMRI for other reasons (myocardial ischemia or myocardial inflammation) within the predefined period, were selected as control subjects instead of healthy volunteers. It was absolutely mandatory that patients as control subjects were only included when CMRI was unremarkable concerning heart size and function, wall motion, valve disease, perfusion, signs of myo- or pericardial inflammation, pericardial and pleural effusion or pulmonary edema, and pulmonary trunk and aortic diameter. Further clinical patient observation confirmed control subject suitability. Contraindications for CMRI and exclusion criteria were: renal failure with a glomerular filtration rate below 30 mL/min/1.73 m^2^, incompatible implants (cochlear or metallic), gadolinium intolerance, claustrophobia, or patient inability.

CMR technique: Standardized imaging was performed at a 1.5 Tesla MRI system (Somatom Avanto, Siemens Healthineers, Forchheim, Germany) using a six-element phased array cardiac coil. The CMRI protocol contained thoracic survey images, CINE sequences, steady-state-free precession sequences (SSFP) aligned to short-axis, 2-, 3- and 4-chamber view (SA, 2-CV, 3-CV and 4-CV), T2-wighted imaging (“black-blood” T2 turbo spin echo), LGE imaging (T1 gradient echo with inversion recovery) and native T1-mapping. Gadoteridol (Gd-HP-DO3A; ProHance, BRACCO Imaging, Milan, Italy) was injected at a dose of 0.15 mmol/kg. LGE imaging was performed 12 min after contrast media injection. SSFP and CINE images were obtained during breath hold. The LV volume (absolute values) was calculated from short-axis CINE images. Measurements were performed on end-diastolic images and end-systolic images. Endocardial contours of the LV were obtained by manual tracing with exclusion of papillary muscles and trabeculae from the cavity. Ventricular volumes were estimated using the Simpson rule. T1 mapping images were acquired at basal, mid-ventricular, and apical short-axis section by using a modified Look-Locker inversion-recovery (MOLLI “3-3-5”) sequence—11 images were acquired during 17 heartbeats, and after in-line motion, correction maps were generated [23]. Imaging parameters for native T1 mapping were: slice thickness: 8 mm; spatial resolution: 2.2 mm × 1.8 mm × 8 mm; 6/8 partial Fourier acquisition; field of view: 240 × 340 mm; matrix: 192 × 124; flip angle 35°; TR 740 and TE 1.06; TI 100 ms and TI Increment 80 ms; trigger delay: 300 ms; inversions 3; acquisition heartbeats: 3-3-5; and scan time: 17 heartbeats. 

CMR analysis: Postprocessing was performed by using the cardiovascular imaging version 42 (cvi42) software including feature and tissue tracking (Circle Cardiovasculare Imaging, Calgary, Alberta, Canada). LV strain was quantified on contiguous SA CINE images (8 to 10 slices on average, depending on patient and cardiac size) and log-axis CINE images aligned to 2-, 3- and 4-CV using the feature and tissue tracking software tool. Global longitudinal (GLS), radial (GRS) and circumferential (GCS) strains were analyzed for the left ventricle and were defined as the peak value of each strain. GLS analysis was performed via 4-CH CINE imaging and GRS or GCS was based on SA CINE imaging at midventricular level.

Septal native T1 times were measured in region of interest (ROI) at basal short-axis section to guarantee proper T1 measurements caused by greater septal myocardial diameters compared to midventricular and apical sections. To avoid measuring of partial volume-averaging artefacts and registration errors with gradual T1 changes at myocardial borders, the ROI were drawn carefully, and software assisted by predefining an epicardial and endocardial offset of 10 percent. All measurements were performed by two experienced radiologists with 10 and 25 years of experience in cardiovascular imaging. LGE assessment was performed blinded to native T1 maps and CINE images. All T1 maps and LGE images were of diagnostic quality and could sufficient be evaluated. 

Intraobserver and interobserver variability were analyzed for septal native T1 mapping and feature tracking strain analysis including GLS, GRS and GCS. Initially, the first investigator (10 years of experience in cardiovascular imaging) performed septal native T1 measurements and feature tracking strain analysis blinded to patient demographics. To assess intraobserver variability, measurements were repeated after a period of 14 days. Moreover, a second experienced investigator (25 years of experience in cardiovascular imaging), who was also blinded to patient demographics, performed septal native T1 measurements and feature tracking strain analysis to determine interobserver variabilities.

Laboratory assessment: Lyso-globotriaosylceramide (LysoGb3) was measured in serum at the (blinded for review). LysoGb3 values > 1.0 ng/mL were interpreted as elevated.

## 3. Statistical Analysis

Statistical analysis was performed using PRISM statistical software version 9 (Graphpad Software, San Diego, CA, USA). Patient characteristics were described by mean ± SD. All data were tested for normal distribution using the Shapiro–Wilk test. In cases of normal distribution, Student’s *t*-test was used, and if the data were not distributed normally, the Mann–Whitney test (non-parametric) was used. Strengths of correlations were tested using the Spearman correlation coefficient. The correlation coefficient r was interpreted according to Hinkle et al. [24] where r > 0.3 would be considered a weak correlation, r > 0.5 would a moderate correlation, r > 0.7 a strong correlation, and r > 0.9 very strong correlation. An r > 0.5 would therefore be considered to have clinical impact. To assess intraobserver and interobserver agreement intra-class concordance correlation coefficient (ICC) was used. An excellent agreement was defined as ICC > 0.8. All results were tested at a 5% level of significance and alpha error of less than 0.05 was accepted as statistically significant.

## 4. Results

Table 1 presents patient and control subject demographics. The mean age of the FD patients was 49.9 years ± 16.8 standard deviation (± SD) and the mean age of the control subjects was 47.5 years ± 13.2 SD. Both, FD patients and control subjects showed normal left heart function with normal left ventricular ejection fraction (LVEF). LVEF was 65.9% ± 8.1 SD (range, 44 to 79%) for FD patients and 67.7% ± 7.9 SD (range, 58 to 78%) for control subjects. End diastolic volume (EDV) and stroke volume (SV) revealed normal values for FD patients and control subjects but EDV and SV were significantly higher for the control subjects (*p* = 0.025 and *p* = 0.026), whereas end systolic volume (ESV) differed not significantly. Intra- and interobserver variability were excellent for LVEF and for all measured cardiac volumes (EDV, ESV and SV) in FD patients and control subjects (r between 0.9488 and 0.9823).

LVH, defined as a septal myocardial diameter equally or greater than 13 mm in diastole, was present in six FD patients (21.4%) and in none of the control subjects. Septal diameter and myocardial mass did not differ significantly between FD patients and control subjects. The mean septal myocardial diameter was 9.8 mm ± 3.6 SD for FD patients and 8.4 m ± 1.6 SD for control subjects and the mean LV myocardial mass was 123.1 g ± 55.5 SD for FD patients and 103.6 g ± 30.0 SD for control subjects. 

The mean septal native T1 time for the FD patients was 921.1 ms ± 49.4 SD (range, 820 to 989 ms) and was statistically significant lower (*p* = 0.0064) compared to the control subjects, who had a mean native septal T1 time of 951.0 ms ± 47.3 SD (range, 917 to 985 ms). LGE at the inferolateral wall was present in seven of the FD patients (25%) and in none of the control subjects.

With exception of GCS (−19.9% ± 3.2 versus −21.6% ± 2.7 SD; *p* = 0.1218), FD patients had significant GLS and GRS reductions compared to the control subjects: GLS −18.0% ± 3.3 versus −20.5% ± 1.7 SD (*p* = 0.0009), GRS 33.6% ± 8.7 versus 41.4% ± 11.0 SD (*p* = 0.0184). Intra- and interobserver variability were excellent for septal native T1 time in FD patients (r = 0.9836, 95% CI 0.9723–0.9898; r = 0.9788, 95% CI 0.9684–0.9865) and for control subjects (r = 0.9812, 95% CI 0.9722–0.9912; r = 0.9815, 95% CI 0.9675–0.9888), for GLS (r = 0.8774, 95% CI 0.8627–0.8956; r = 0.8698, 95% CI 0.8488–0.8967) and (r = 0.8856, 95% CI 0.8698–0.9012; r = 0.8731, 95% CI 0.8643–0.8866), GRS (r = 0.8626, 95% CI 0.8456–0.8844; r = 0.8598, 95% CI 0.8412–0.8729) and (r = 0.8688, 95% CI 0.8522–0.8810; r = 0.8602, 95% CI 0.8468–0.8798), GCS (r = 0.8512, 95% CI 0.8366–0.8788; r = 0.8688, 95% CI 0.8422–0.8866) and (r = 0.8498, 95% CI 0.8343–0.8688; r = 0.8523, 95% CI 0.8388–0.8652).

**Figure 1:**
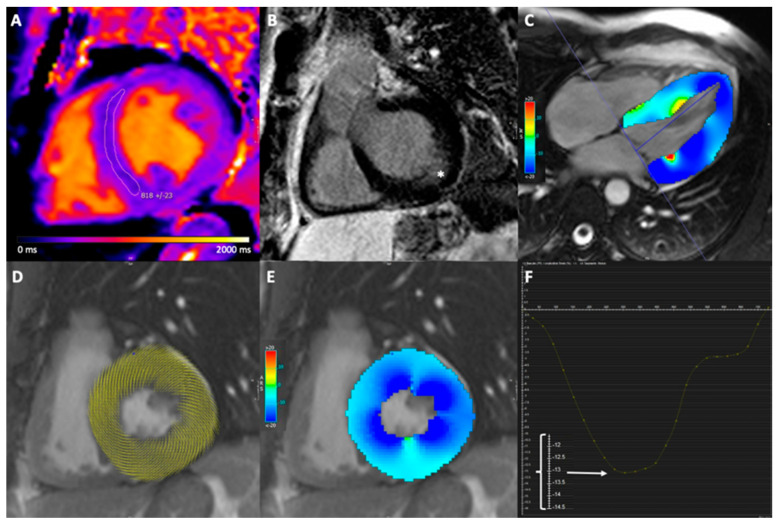
Imaging example including native T1 map, LGE image and strain images. Cardiovascular magnetic resonance images of a 38-year-old male with Fabry disease. Midventricular short-axis native T1 map (**A**) demonstrates reduced septal native T1 time, and basal short-axis late gadolinium enhanced (LGE) image (**B**) demonstrates intramural LGE at the inferior lateral wall (white asterisk). Short-axis CINE steady state free precession (SSFP) image with circumferential myocardial strain analysis points (**D**) and color-coded myocardial circumferential strain map (**E**). Long-axis CINE SSFP image with color-coded myocardial longitudinal strain map (**C**). Global longitudinal strain was −13.2%, as the enlarged scale on the Y-axis showed (**F**).

**Figure 2:**
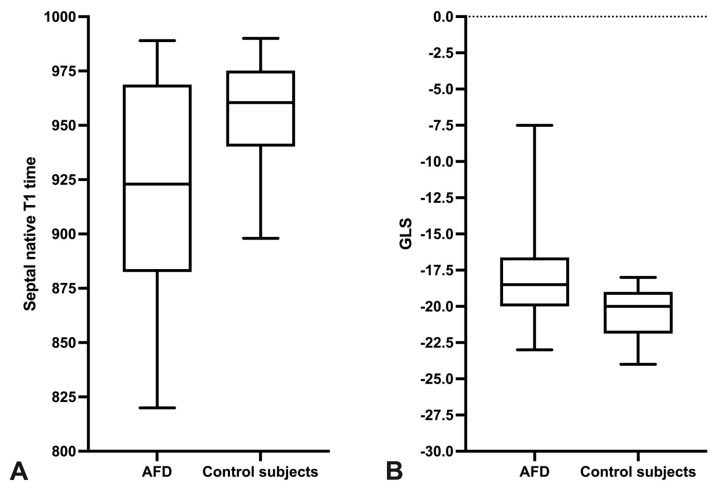
Septal native T1 times and GLS in FD patients and control subjects. The boxplots present the septal native T1 times (**A**) and the global longitudinal strain (GLS) (**B**) in FD patients and in control subjects.

Figure 1 shows native T1 and strain measurements in an FD patient and Figure 2 shows the boxplots for septal native T1 times and GLS in FD patients and control subjects. Cardiac function, cardiac morphology and strain values for FD patients and control subjects are presented in Table 2.

Correlation of septal native T1 times, strain values and LysoGb3: The mean LysoGb3 level of the FD patients was 17.8 ng/mL ± 38.1 SD (range, 0.3 to 169). Septal native T1 time and GLS revealed the best correlations to LysoGb3 with a significant moderate positive correlation for GLS and LysoGb3 (r = 0.5498; *p* = 0.0024) and a significant negative moderate correlation for septal native T1 times and LysoGb3 (r = −0.6519; *p* = 0.0002). All correlations are presented in Table 3.

**Figure 3:**
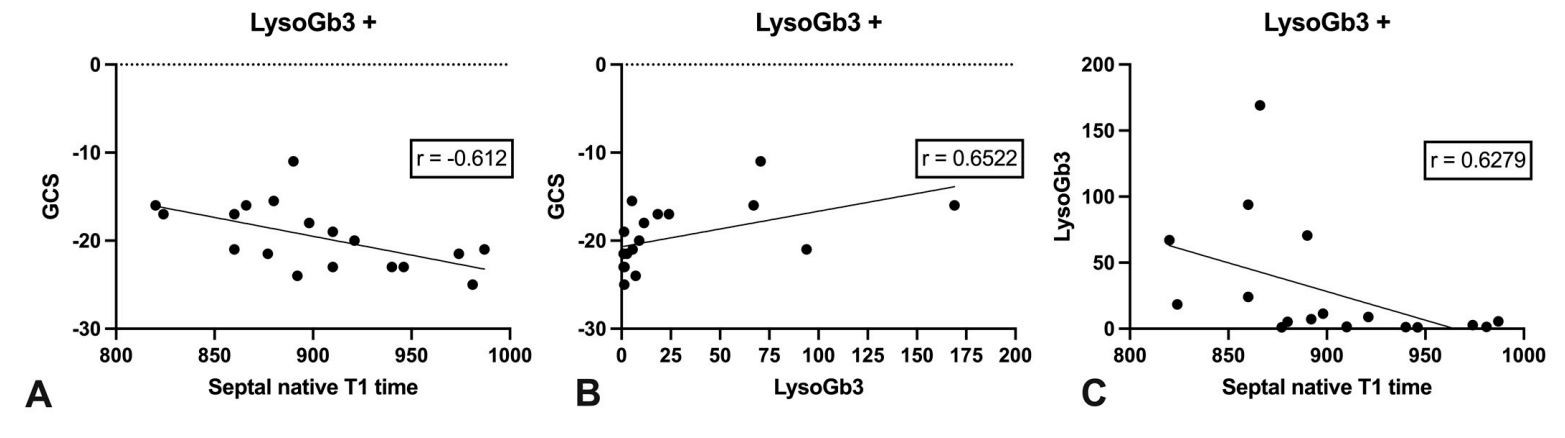
Correlations of LysoGb3 positive FD patients to GCS and septal native T1 time. Scatter plots showing the correlations of GCS and septal native T1 time (**A**), GCS and LysoGb3 (**B**), and septal native T1 time and LysoGb3 (**C**) in LysoGb3 positive (+) FD patients.

LysoGb3 positive and LysoGb3 negative FD patients: LGE was only present in LysoGb3 positive FD patients. Cardiac function, septal diameter and myocardial mass differed not significantly between LysoGb3 positive and LysoGb3 negative FD patients, whereas septal native T1 times and GLS were significantly reduced in LysoGb3 positive FD compared to LysoGb3 negative patients (*p* = 0.005 and *p* = 0.03). Cardiac function, cardiac morphology and strain values for LysoGb3 positive and negative FD patients are presented in Table 4. Continuously, a moderate negative correlation for septal native T1 times and LysoGb3 in the LysoGb3 positive patients was present (r = −0.6279; *p* = 0.0053). Apart from the correlation of septal native T1 times to GLS all other correlations of septal native T1 times, strain values (GCS, GRS) were increased in LysoGb3 positive patients compared to all FD patients. Correlations are presented in Table 5 and Figure 3. Thereby, GCS showed the best correlations to septal native T1 times and LysoGb3 with a significant negative moderate correlation for GCS and septal native T1 times (r = −0.612; *p* = 0.0069) and a significant moderate correlation for GCS and LysoGb3 (r = 0.6522; *p* = 0.034).

**Figure 4:**
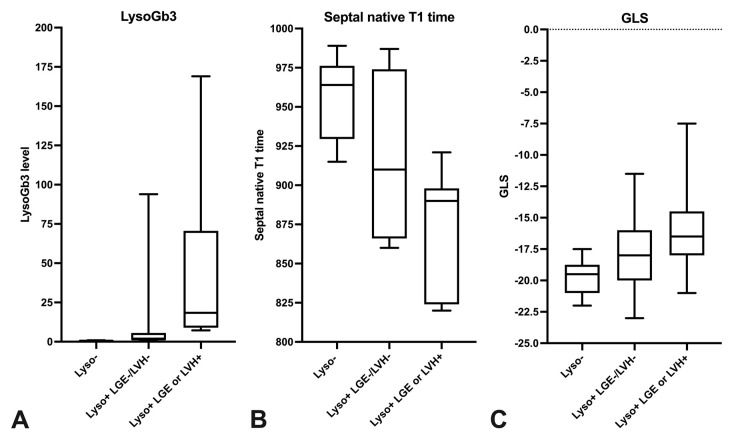
FD patient subgroup analysis. The boxplots present the LysoGb3 level (**A**), the septal native T1 times (**B**) and the global longitudinal strain (GLS) (**C**) of FD patients depending on LysoGb3 positivity and presence of late gadolinium enhancement (LGE) or left ventricular hypertrophy (LVH).

**Figure 5:**
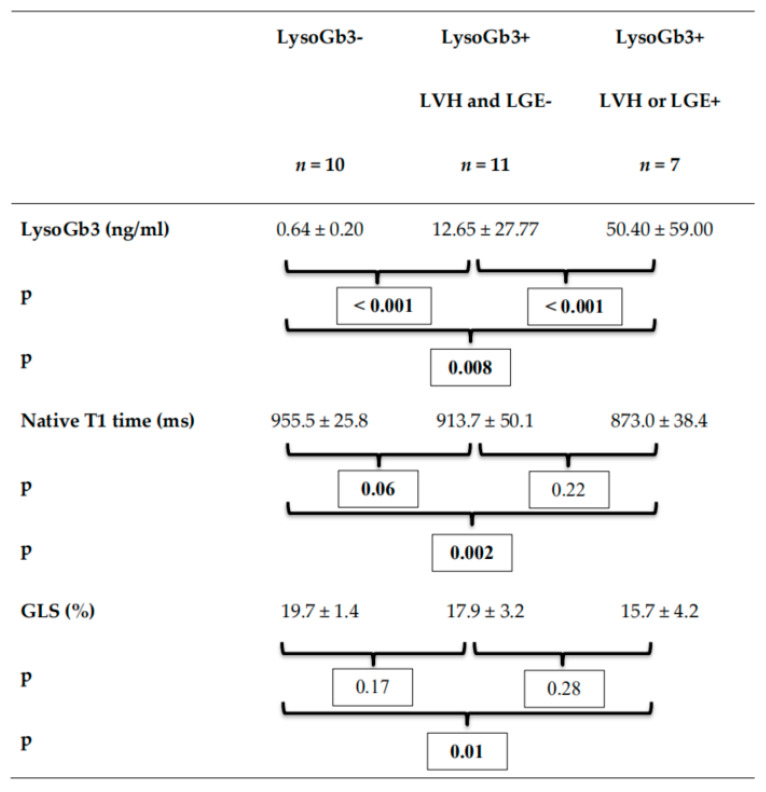
LysoGb3, septal native T1 and GLS in FD subgroups. GLS—global longitudinal strain, *p*—significance value.

Moreover, FD subgroup analysis between LysoGb3 negative FD patients without LGE and LVH, LysoGb3 positive FD patients without LGE or LVH and LysoGb3 positive patients with either LGE or LVH showed increasing and significantly different LysoGb3 levels between all groups and significant reductions of septal native T1 times between LysoGb3 negative, and both LysoGb3 positive patient groups and a significant reduction in GLS between LysoGb3 negative FD patients and LysoGb3 positive patients with either LGE or LVH. Subgroup FD analysis is presented in Figure 4 via boxplots and in Figure 5.

Key points:

Native T1 times are significantly decreased in FD.
Feature tracking strain values are also decreased in FD.
Native T1 times and strain values showed promising correlations to biomarker LysoGb3.
Moreover, GLS and native T1 times reduce with LysoGb3 increase.
Mechanical dysfunction may occur early in FD.

## 5. Discussion

In the last decade, CMRI has been changed from semiquantitative assessment to quantitative phenotyping. The mainly descriptive imaging with visual assessment of edema, hyperemia, LGE and wall motion abnormalities has changed to quantitative functional and morphologic imaging due to rapid technical development.

Hereby, improved disease burden quantification, therapy monitoring, follow-up and prognosis estimation is enabled in many cardiac diseases. In the course of FD, especially native T1 mapping showed great potential for detection of early cardiac involvement [12,13,14] as myocardial storage and accumulation of glycosphingolipids shorten the native T1 time.

In contrast to FD, other cardiomyopathies show native T1 time elevations and cardiac FD involvement can therefore be proven well independently of LVH [12]. Reductions of native T1 times in FD are present in absence of LVH, and diagnosis of early cardiac manifestation is possible prior to development of typical (“hallmark”) LGE at the inferolateral wall. Hence, the most appropriate patients for ERT may be detectable via native T1 mapping.

FT-SA showed promising results in different cardiac diseases [20,21], but data on CMR derived FT-SA in FD is still limited. Besides previous performed echocardiographic studies, which showed strain abnormalities in FD patients [16,17,18,19], first CMRI studies dealing with FT-SA in FD patients showed promising results. Based on their results with association of impairment in GLS and reduction of native T1 times without presence of LVH, the authors of a recent study suggested that mechanical dysfunction in the course of FD occurs before evidence of glycosphingolipid deposition [25]. Another study concluded that base to apex circumferential strain may be an early marker of cardiac FD involvement with independent and incremental value beyond native T1 [26].

The goal of our study was to investigate the relations of FT-SA to native T1 times and to the FD specific biomarker LysoGb3.

The main findings of our study are:

1. Septal native T1 times, GLS and GRS are significantly reduced in FD patients compared to control subjects, whereas cardiac function (LVEF), septal diameter and myocardial mass differed not significantly. Moreover, septal native T1 times and GLS showed moderate correlations to LysoGb3 (FD specific biomarker);
2. Compared to LysoGb3 negative FD patients, LysoGb3 positive FD patients had significantly reduced septal T1 times. Furthermore, correlations of strain values with LysoGb3 and septal native T1 times increased in LysoGb3 positive FD patients.
3. Finally, subgroup analysis between 1. LysoGb3 negative FD patients, 2. LysoGb3 positive FD patients without LVH or LGE and 3. LysoGb3 positive FD patients with either LVH or LGE showed a steady decrease of septal native T1 time and GLS accompanied by a steady increase of LysoGb3. Hence, GLS initially reduces with storage (low native T1 times and elevated LysoGb3 level) and later with increasing LysoGb3 level and hypertrophy or scar (LGE).

Consistent with the results of previously published studies [12,13,14], the native T1 times were significantly reduced in our FD patients. Moreover, the significantly reduced strain values in our FD patients also support the findings of the previous published studies [25,26]. To the best of our knowledge, this is the first study in the field that investigated the relations of FT-SA values with native T1 times and the FD specific biomarker LysoGb3. LysoGb3 plasma levels are higher in all subgroups of FD than in healthy subjects and tend to be high in FD patients with proceeded disease stages inter alia in patients with developed heart disease [15].

The main limitation of our study is the small number of patients. Otherwise, it is a prospective cohort analysis in a rare disease with untreated patients, and the results of the FD subgroup analysis revealed significant differences regarding native T1 times and FT-SA values. Our results may therefore serve as a starting point for further investigations assessing biomarkers, native T1 times and strain values in the course of FD.

## 6. Conclusions

The steady GLS and native T1 decrease from LysoGb3 negative FD patients over LysoGb3 positive FD patients to LysoGb3 positive FD patients with presence of LVH or LGE and the promising correlations of strain values, native T1 times and LysoGb3 suggests that wall motion abnormalities occur early in cardiac FD involvement. We therefore suggest a diagnostic cardiac MRI algorithm in FD containing FT-SA beside native T1 measurements and LysoGb3 analysis to enable best possible early cardiac disease manifestation detection with the goal to avoid unfavorable disease progression due to early initiation of ERT. 1. LysoGb3 elevation and native T1 or FT-SA decrease—cardiac FD manifestation seems to be likely, 2. LysoGb3 elevation with both, native T1 and FT-SA decrease—cardiac FD manifestation seems likely, 3. LysoGb3 elevation with normal native T1 and FT-SA values—cardiac FD manifestation remains unclear and close controls should be performed.

## Figures and Tables

**Table 1:** Baseline characteristics.

	FD	Control Group
Patients (*n*)	28	28
Sex (male:female)	10:18	10:18
Age ± SD (years)	49.9 ± 16.8	47.5 ± 13.2
Lyso-Gb3 (ng/mL)	17.8 ± 38.1	

Values are mean ± SD or absolute values.

**Table 2:** Cardiac function and morphology: FD versus control group.

	FD *n* = 28	Control Group *n* = 28	*p*
LVEF (%)	65.9 ± 8.1	67.7 ± 7.9	0.68
EDV (mL)	108.5 ± 32.4	125.9 ± 30.1	0.025
ESV (mL)	37.5 ± 14.0	41.8 ± 16.5	0.32
SV (mL)	72.1 ± 21.3	84.1 ± 17.4	0.026
Septal diameter (mm)	9.8 ± 3.6	8.4 ± 1.6	0.266
Myocardial mass (g)	123.1 ± 55.5	103.6 ± 30.0	0.189
Septal native T1 time (ms)	921.1 ± 49.4	951.0 ± 47.3	0.006
GRS (%)	33.6 ± 8.7	41.4 ± 11.0	0.018
GCS (%)	−19.9 ± 3.2	−21.6 ± 2.7	0.122
GLS (%)	−18.0 ± 3.3	−20.5 ± 1.7	<0.001
LGE (*n*; %)	7 (25%)	-	

Values are mean ± SD, LVEF—left ventricular ejection fraction, EDV—end diastolic volume, ESV—end systolic volume, SV—stroke volume, GRS—global radial strain, GCS—global circumferential strain, GLS—global longitudinal strain, LGE—late gadolinium enhancement, *p*—significance value.

**Table 3:** Correlations of strain values, LysoGb3, myocardial mass and native T1.

	r	95% CI	*p*
Septal native T1 to GRS	0.3272	−0.06385 to 0.6311	0.089
Septal native T1 to GCS	−0.4687	−0.7221 to −0.1044	0.012
Septal native T1 to GLS	−0.3251	−0.6297 to −0.06620	0.092
LysoGb3 to GRS	−0.288	−0.6028 to 0.1091	0.14
LysoGb3 to GCS	0.384	0.00114 to 0.6687	0.044
LysoGb3 to GLS	0.5498	0.2114 to 0.7706	0.002
Septal native T1 to LysoGb3	−0.6519	−0.8281 to −0.3584	<0.001

GRS—global radial strain, GCS—global circumferential strain, GLS—global longitudinal strain, r—correlation coefficient, CI—confidence interval, *p*—significance value.

**Table 4:** Cardiac function and morphology: FD Lyso-Gb3+ versus Lyso-Gb3-.

	LysoGb3+ *n* = 18	LysoGb3− *n* = 10	*p*
LVEF (%)	65.9 ± 8.8	66.1 ± 6.9	0.98
EDV (mL)	111.5 ± 34.1	103.0 ± 30.0	0.86
ESV (mL)	38.4 ± 16.3	35.8 ± 9.1	0.98
SV (mL)	73.1 ± 22.5	70.3 ± 20.0	0.76
Septal diameter (mm)	10.7 ± 4.1	8.0 ± 1.3	0.059
Myocardial mass (g)	135.4 ± 64.1	96.8 ± 25.5	0.10
Septal native T1 time (ms)	902.0 ± 49.4	955.5 ± 25.8	0.005
GRS (%)	32.2 ± 10.0	36.3 ± 5.1	0.41
GCS (%)	−19.6 ± 3.7	−20.5 ± 2.3	0.58
GLS (%)	−17.0 ± 3.7	−19.7 ± 1.4	0.03
LGE (*n*; %)	7 (70%)	0	

Values are mean ± SD, LVEF—left ventricular ejection fraction, EDV—end diastolic volume, ESV—end systolic volume, SV—stroke volume, GRS—global radial strain, GCS—global circumferential strain, GLS—global longitudinal strain, late gadolinium enhancement, *p*—significance value.

**Table 5:** Correlations of strain values, LysoGb3 and native T1 in LysoGb3+ patients.

	r	95% CI	*p*
Septal native T1 to GRS	0.4410	−0.04752 to 0.7593	0.067
Septal native T1 to GCS	−0.612	−0.8435 to −0.1889	0.007
Septal native T1 to GLS	−0.394	−0.7370 to 0.09780	0.101
LysoGb3 to GRS	−0.4708	−0.7747 to 0.009960	0.049
LysoGb3 to GCS	0.6522	0.2524 to 0.8617	0.034
LysoGb3 to GLS	0.4602	−0.02347 to 0.7693	0.043
Septal native T1 to LysoGb3	−0.6279	−0.8508 to −0.2136	0.005

GRS—global radial strain, GCS—global circumferential strain, GLS—global longitudinal strain, r—correlation coefficient, CI—confidence interval, *p*—significance value.

## Data Availability

The datasets used or analyzed during the current study are available from the corresponding author on reasonable request.

## Abbreviations

FD	Fabry Disease
CMRI	cardiac magnetic resonance imaging
CV	chamber view
FT-SA	feature tracking strain analysis
EDV	end diastolic volume
ESV	end systolic volume
ERT	enzyme replacement therapy
GCS	global circumferential strain
GLS	global longitudinal strain
GRS	global radial strain
LGE	late gadolinium enhancement
LV	left ventricular
LVEF	left ventricular ejection fraction
LVH	left ventricular hypertrophy
ROI	region of interest
SA	short axis
SD	standard deviation
SSFP	steady-state-free precession
SV	stroke volume
